# The Compliance of Current Small Animal CPR Practice With RECOVER Guidelines: An Internet-Based Survey

**DOI:** 10.3389/fvets.2019.00181

**Published:** 2019-06-11

**Authors:** Íde Gillespie, Daniel J. Fletcher, Mark A. Stevenson, Manuel Boller

**Affiliations:** ^1^Faculty of Veterinary and Agricultural Sciences, Melbourne Veterinary School, University of Melbourne, Werribee, VIC, Australia; ^2^Department of Clinical Sciences, College of Veterinary Medicine, Cornell University, Ithaca, NY, United States

**Keywords:** dog, cat, cardiac arrest, cardiopulmonary resuscitation, heart, guidelines

## Abstract

In 2012 the Reassessment Campaign on Veterinary Resuscitation (RECOVER) published evidence-based treatment recommendations for dogs and cats with cardiopulmonary arrest (CPA), to optimize the clinical practice of small animal CPR and positively impact outcomes. Six years after the release of these guidelines, we aimed to determine the compliance of small animal veterinary CPR practices with these RECOVER guidelines. To identify current CPR practices in clinically active small animal veterinarians and their awareness of the RECOVER guidelines, we conducted an internet-based survey. Survey invitations were disseminated internationally via veterinary professional organizations and their social media outlets. Questions explored respondent demographics, CPR preparedness, BLS and ALS techniques and awareness of RECOVER guidelines. Responding small animal veterinarians (*n* = 770) in clinical practice were grouped by level of expertise: board-certified specialists (BCS, *n* = 216) and residents (RES, *n* = 69) in anesthesia or emergency and critical care, practitioners in emergency (GPE, *n* = 299) or general practice (GPG, *n* = 186). Large disparities in preparedness measures, BLS and ALS techniques emerged among levels of expertise. Only 32% (95% CI: 29–36%) of respondents complied with BLS practice guidelines, varying from 49% (95% CI: 42–55%) of BCS to 15% (95% CI: 10–20%) of GPG. While incompliances in BCS, RES, and GPE were predominantly due to knowledge gaps, GPG compliance was further compromised by limitations in the resuscitation environment (e.g., defibrillator availability, team size). Those aware of RECOVER guidelines (100% of BCS and RES; 77% of GPE; 35% of GPG) were more likely to comply with recommended preparedness (OR = 2.4; 95% CI: 1.2–4.8), BLS (OR = 4.5; 95% CI: 2.4–9.1), and ALS techniques (OR = 7.8; 95% CI: 2.4–9.1) independent of age, gender, region of practice or level of expertise. We conclude that awareness of RECOVER guidelines is high in specialists and residents, but incomplete among general practitioners. This awareness positively influenced compliance with CPR guidelines, but CPR practices continue to be variable and largely not in agreement with guidelines. A widely accessible educational strategy is required to broadly improve compliance with best practices in small animal CPR.

## Introduction

Mortality is extremely high in small animals experiencing in-hospital cardiopulmonary arrest (CPA). Survival to discharge rates range from 1.6–6% in dogs, and 2.3–9.6% in cats (1–5), whereas in humans, approximately 12% and 24% of adults survive out-of-hospital and in-hospital cardiac arrest, respectively (6). Implementation of a comprehensive CPR strategy, starting with recognition of CPA risk factors and extending far into the post-resuscitative phase, is imperative to improve survival rates associated with CPA (6, 7).

Results of an internet-based survey conducted in 2008 and published in 2010 evaluated the clinical practice of veterinary CPR. This study concluded that CPR was heterogeneously performed in small animal veterinary medicine (8). In 2012, the Reassessment Campaign on Veterinary Resuscitation (RECOVER) initiative published the first evidence-based, consensus CPR guidelines for small animals. The RECOVER initiative systematically evaluated CPR literature, developed clinical guidelines on optimal CPA treatment in dogs and cats, and identified important knowledge gaps (9, 10). These guidelines have now been largely adopted by the veterinary emergency and critical care (ECC) community as standard of care in CPR as they are the foundation for most CPR textbook chapters, are part of ECC specialty training, are endorsed by the American College of Veterinary Emergency and Critical Care (ACVECC) and the Veterinary Emergency and Critical Care Society (VECCS) and are widely used in training and certification of veterinary professionals in CPR (11–16). The question now arises to what extent current CPR practices comply with these guidelines, and if the level of compliance is affected by respondent awareness of the RECOVER guidelines. The presence of substantial incompliance with RECOVER guidelines would indicate that efforts need to be increased to improve guideline uptake in the veterinary community. Moreover, a better understanding of the nature of compliances in different professional environments will provide the foundation to develop targeted strategies to increase adoption of best practice in CPR and to improve patient outcome.

To address these questions, we analyzed the data from an international, internet-based survey that broadly examined the clinical practice in small animal CPR. The objectives of our study were to determine the agreement of CPR practices of clinically active small animal veterinarians with current guidelines and to quantify the association of RECOVER awareness with CPR guidelines compliance.

## Materials and Methods

We used an anonymous online survey to interview clinically active small animal veterinary professionals on their clinical practice of CPR after the University of Melbourne's Human Ethics Committee approved the survey procedure (Ethics ID 1546061). From the survey data that encompassed all aspects of small animal CPR, we extracted the questions that describe respondent characteristics, those that reflect specific RECOVER guidelines and those that concern respondent awareness of the RECOVER guidelines. The entirety of descriptive survey data on the current practice in veterinary CPR is published elsewhere.

### Data Collection

Survey invitations were distributed internationally via electronic mailing lists of various professional veterinary organizations and their social media outlets. These organizations were the American College of Veterinary Anesthesia and Analgesia (ACVAA; 771 emails), the Veterinary Emergency and Critical Care Society (VECCS; 4,427 emails), American College of Emergency and Critical Care (ACVECC; diplomats, 596 emails; residents, 175 emails; residency trained, 60 emails), the European Veterinary Emergency and Critical Care Society (EVECCS; 466 emails), the Australian and New Zealand College of Veterinary Scientists (ANZCVS; 2,095 emails), and the Academy of Veterinary Emergency and Critical Care Technicians (AVECCT; 450 emails). The initial emails containing invitations to take part in the survey were sent on March 1, 2017, followed by reminder emails sent on March 8 and 15. The survey closed on March 31. Survey links were also made available on the social media outlets of the above organizations over the same time period. Neither the email invitations nor the text used in social media postings included the term RECOVER.

We used REDCap (Research Electronic Data Capture), a software toolset for electronic collection and management of research and clinical trial data, to implement the online survey (17).

### Survey and Population Characteristics

The survey posed 36 questions regarding respondent and patient demographics, CPR statistics such as training, preparedness, equipment, drugs and technique, as well as opinions on CPR skills and RECOVER guidelines (Supplementary Material). Based on a pre-release survey evaluation pilot study, respondents were expected to complete the survey in approximately 10 min. From this large survey relating to all aspects of clinical practice in CPR, we here focused on those questions that pertain to the objectives of this study.

The design of the survey only permitted those respondents indicating that they were currently providing small animal (dogs and/or cats) clinical veterinary care, access to the survey questionnaire. The initial 14 questions surveyed population characteristics, such as gender, age, current career status, and working environment. We used these questions to apply inclusion and exclusion criteria. Responses from non-veterinarians (e.g., technicians and students) and residents or specialists in areas other than anesthesia (ECVAA, ACVAA, or FANZCVSc in anesthesia), or emergency and critical care (ACVECC, ECVECC, or FANZCVSc in ECC) were excluded from analysis. Furthermore, we excluded partially completed surveys. We then divided the remaining records into four groups based on the qualifications of the individuals completing the survey, namely board-certified specialists (BCS), residents (RES), general practitioners working as emergency veterinarians (GPE), and general practitioners working in non-emergency positions (GPG). BCS and RES included respondents that were board-certified or residents, respectively, in veterinary emergency and critical care or in anesthesia. GPG referred to non-BCS veterinarians working in general practice offering an emergency service during business hours with or without an after-hours on-call service. Non-BCS veterinarians working in an after-hours emergency clinic, a 24 h emergency clinic, or an emergency/critical care center, were designated group GPE. Interns were allocated to either GPG or GPE using the same criteria.

Eleven questions contained information relevant for CPR compliance in the areas of preparedness, BLS and ALS. An additional three questions at the end of the survey inquired on respondents' awareness of the 2012 RECOVER consensus guidelines and their opinion regarding the usefulness of the guidelines to improve CPR.

### Compliance With CPR Guidelines

Based on the raw survey data we classified a respondent as compliant in each area of interest (i.e., preparedness, BLS, and ALS) when the respondent's answers were in agreement with the RECOVER guidelines. Compliance in preparedness was defined as cognitive aids (i.e., dosing chart and CPR algorithm) being displayed and a regularly maintained crash cart being present in the practice, as well as having participated in CPR training within the last 6 months. Respondents who selected the recommended chest compression (i.e., 100–120 compressions per minute [cpm]) and ventilation rates (i.e., 6–15 breaths per minute), for both dogs and cats were deemed BLS compliant per the RECOVER guidelines. We considered respondents with access to a defibrillator, routinely using ECG and ETCO_2_ but not intravascular volume expansion, and access to CPR drugs with a level I, IIa, or IIb recommendation in the RECOVER guidelines (epinephrine or vasopressin, atropine, amiodarone or lidocaine, and sodium bicarbonate) ALS compliant (10).

### Statistical Analyses

We exported survey data from REDCap into a commercial spreadsheet computer program^1^ and subsequently to a commercial statistical software package^2^ for further analysis. We used standard descriptive statistics to summarize continuous and categorical data, with measures of central tendency and spread quantified, as appropriate. The normality of continuous data was tested with the Shapiro-Wilk test. Group response rates were presented as crude proportions (i.e., not adjusted for the influence of potential confounders) with 95% confidence intervals in table or bar charts. When comparing response proportions of more than two groups, we used analysis of variance or Pearson's Chi Square tests to identify significant differences amongst groups. The Student's *t*-test (for continuous data) or Fisher's exact test (for categorical data) were used for comparisons of responses among two groups.

Individual respondent-level variables that were likely to confound the association between compliance in a specific CPR domain (i.e., preparedness, BLS, and ALS) and awareness of RECOVER guidelines, were controlled-for using binomial logistic regression. These variables were professional group (e.g., BCS vs. RES), gender, age, and region of work. The effect of each variable on compliance was expressed as odds ratios and we used the likelihood-ratio test and associated *P*-values for hypothesis testing. The significance level was set at an alpha value of 0.05.

## Results

### Respondent Characteristics

**Figure 1:**
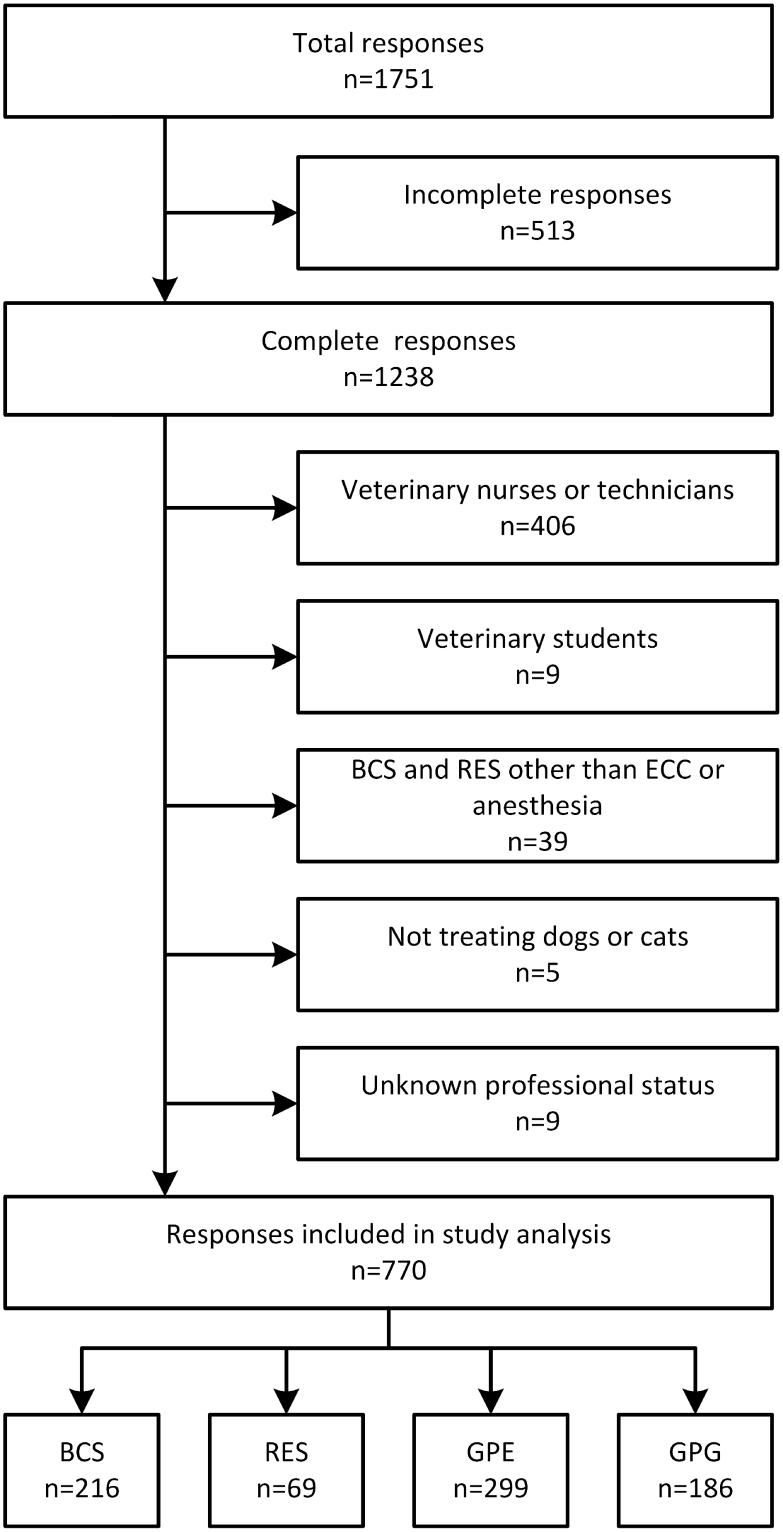
Flow diagram of respondent identification and inclusion into final analysis. BCS, board-certified specialists in emergency and critical care and/or anesthesia; RES, residents in anesthesia or emergency and critical care; GPE, general practitioners working in emergency environment; GPG, general practitioners in non-emergency environment; ECC, emergency and critical care.

Of the 1,751 survey participants, we excluded 981 responses because of submission by non-target responders or incompleteness (Figure 1). Data from non-target responders (e.g., veterinary nurses or technicians) will be reported in a separate scientific communication. Of the incomplete responses, 22 were in BCS, 16 in RES, 70 in GPE, and 63 in GPG, the remainder in non-target responders. The remaining 770 records were comprised of 216 (28%) BCS, 69 (9%) RES, 299 (39%) GPE, and 186 (24%) GPG (Table 1). There was no gender difference between groups (*P* = 0.09). The mean age of all respondents was 38 ± 9 years with a significant age difference between BCS and RES (*P* < 0.0001) (Table 2). Residents and BCS attended to a significantly higher proportion of emergencies (more than 50% of caseload) compared to 7% of GPG (*P* < 0.0001). Forty-eight percent of RES (95% CI: 37–59%), 38% of BCS (95% CI: 32–45%), and 34% of GPE (95% CI: 28–40%) indicated that they were directly involved in CPR more than 20 times per year, while GPG performed CPR predominantly once (31%; 95% CI: 25–38%) or 2–5 times (41%; 95% CI: 35–49%) per year. There was a significant difference in resuscitation team size between groups. Almost all BCS and RES and the majority of GPE performed CPR in teams of 4 or more, whereas GPG had predominantly 1 or 2 team members (45%; 95% CI: 38–52%) or 3 team members (37%; 95% CI: 30–44%) available (Table 2).

**Table 1:** Emergency setting of respondents' practices across different groups.

	**All groups**	**BCS**	**RES**	**GPE**	**GPG**	***P*-value**
Emergency service during regular business hours only	108 [14, 12–17]	1 [1, 0–3]	1 [2, 0–8]	0 [0, NA]	106 [57, 50–64]	<0.0001
Emergency service during regular business hours and on call service after hours	89 [12, 9–14]	10 [5, 3–8]	1 [2, 0–8]	0 [0, NA]	78 [42, 35–49]	<0.0001
Emergency clinic or service open after regular business hours only	40 [5, 4–7]	4 [2, 1–5]	1 [2, 0–8]	35 [12, 9–16]	0 [0, NA]	<0.0001
Emergency clinic or service open 24 hours and able to hospitalize patients	222 [29, 26–32]	33 [15, 11–21]	13 [19, 11–30]	176 [59, 53–64]	0 [0, NA]	<0.0001
Emergency/critical care center with board certified emergency clinicians and technicians	303 [39, 36–43]	166 [77, 71–82]	53 [77, 66–85]	84 [28, 23–33]	0 [0, NA]	<0.0001
University practice	178 [23, 20–26]	89 [41, 35–48]	49 [71, 59–80]	28 [9, 7–13]	12 [7, 4–11]	<0.0001

*BCS, board-certified specialists in emergency and critical care and/or anesthesia; RES, residents in emergency and critical care and/or anesthesia; GPE, general practitioners in emergency practice; GPG, general practitioners in non-emergency environment; NA, non-applicable. Number of respondents is expressed as count [percentage of group total, 95% confidence interval]. P-value refers to differences among all groups*.

**Table 2:** Population characteristics of groups of respondents.

**Characteristic**	**All groups**	**BCS**	**RES**	**GPE**	**GPG**	***P*-value**
Number of respondents	770 [100, NA]	216 [28, 25–31]	69 [9, 7–11]	299 [39, 35–42]	186 [24, 21–27]	<0.0001
Age [years, mean ± SEM]	37.7 ± 0.3	41.1 ± 0.6	31.9 ± 1.0	37.4 ± 0.5	36.6 ± 0.6	<0.0001
Female respondents	554 [72, 69–75]	160 [74, 68–80]	54 [78, 67–86]	219 [74, 68–78]	121 [65, 58–72]	0.09
Number of veterinarians in the practice [median, first (Q1) and third quartile (Q3)]	12, Q1 = 5 Q3 = 28	25, Q1 = 15 Q3 = 50	46, Q1 = 20 Q3 = 60	10, Q1 = 6 Q3 = 20	3, Q1 = 2 Q3 = 5	<0.0001
Respondents with caseload consisting of more than 50% emergencies	408 [53, 49–56]	137 [63, 57–70]	45 [65, 53–75]	214 [72, 66–76]	12 [7, 4–11]	<0.0001
Respondents that are personally performing CPR 6 or more times/year	476 [62, 58–65]	170 [79, 73–84]	54 [78, 67–86]	213 [71, 66–76]	39 [21, 16–27]	<0.0001
Respondents with size of resuscitation team of 4 or more.	458 [60, 56–63]	193 [89, 85–93]	63 [91, 82–96]	167 [56, 50–61]	35 [19, 14–25]	<0.0001

*BCS, board-certified specialists in emergency and critical care and/or anesthesia; RES, residents in emergency and critical care and/or anesthesia; GPE, general practitioners in emergency practice; GPG, general practitioners in non-emergency environment; NA, non-applicable. Characteristics are expressed as number of respondents [percentage of group total, 95% confidence interval] unless stated otherwise. P-value refers to comparisons among all groups*.

Forty-seven percent of all respondents practiced in the USA, 24% in Europe, and 15% in Latin America. The majority of BCS (66%), RES (63%), and GPE (51%) respondents were based in the USA. A high proportion of GPG (41%) practiced in Latin America (Table 3).

**Table 3:** Distribution of respondents' region of practice for each group.

**Group**	**USA**	**Canada**	**Latin America**	**Europe**	**Asia**	**ANZ**	**Africa**
All groups	362 [47, 44–51]	31 [4, 3–6]	115 [15, 13–18]	181 [24, 21–27]	14 [2, 1–3]	60 [8, 6–10]	2 [0, 0–1]
BCS	140 [66, 58–71]	18 [8, 5–13]	1 [1, 0–3]	43 [20, 15–26]	1 [1, 0–3]	12 [6, 3–10]	1 [1, 0–3]
RES	43 [62, 51–73]	0 [0, NA]	0 [0, NA]	21 [24, 21–42]	0 [0, NA]	5 [7, 3–16]	0 [0, NA]
GPE	152 [51, 45–56]	10 [3, 2–6]	37 [12, 9–17]	70 [23, 19–29]	5 [2, 1–4]	22 [7, 5–11]	0 [0, NA]
GPG	27 [15, 10–20]	3 [2, 1–5]	77 [41, 35–49]	47 [25, 20–32]	8 [4, 2–8]	21 [11, 8–17]	1 [1, 0–3]

*ANZ, Australia and New Zealand; BCS, board-certified specialists in emergency and critical care and/or anesthesia; RES, residents in emergency and critical care and/or anesthesia; GPE, general practitioners in emergency practice; GPG, general practitioners in non-emergency environment; NA, non-applicable. Number of respondents from each region is expressed as count [percentage of group total, 95% confidence interval]*.

### Compliance With Preparedness Guidelines

The vast majority of BCS, RES, and GPE respondents had emergency drug dosing charts displayed and crash carts available in their practices (Table 4). Among all respondents, an emergency drug dosing chart was more commonly displayed (84%; 95% CI: 81–86%) than a CPR algorithm (59%; 95% CI: 55–62%) (*P* < 0.0001). The latter was most commonly made available in RES' and BCS' institutions, followed by GPE and GPG. Thirteen percent (95% CI: 9–19%) of GPG and 3% (95% CI: 2–6%) of GPE indicated that they did not have any of the suggested CPR preparation measures in place in their practices. More than half of RES (53%; 95% CI: 42–65%) and 48% (95% CI: 41–54%) of BCS had participated in CPR training in the 6 months prior to completing the survey compared with 38% (95% CI: 33–43%) of GPE and 21% (95% CI: 16–28%) of GPG.

**Table 4:** Summary of responses to the question: which of the following preparedness measures for CPR are in place in your practice?

	**All groups**	**BCS**	**RES**	**GPE**	**GPG**	***P*-values**
Regularly maintained crash cart or crash station	625 [81, 78–84]	210 [97, 94–99]	66 [96, 88–99]	252 [84, 80–88]	97 [52, 45–59]	<0.0001
Emergency drugs dosing chart displayed	646 [84, 81–86]	209 [97, 93–98]	66 [96, 88–99]	260 [87, 83–90]	111 [60, 53–66]	<0.0001
CPR algorithm displayed	453 [59, 55–62]	168 [78, 72–83]	58 [84, 74–91]	167 [56, 50–61]	60 [32, 26–39]	<0.0001
Last CPR training <6 month ago	293 [38, 35–42]	103 [48, 41–54]	37 [53, 42–65]	113 [38, 33–43]	40 [21, 16–28]	<0.0001

*BCS, board-certified specialists in emergency and critical care and/or anesthesia; RES, residents in emergency and critical care and/or anesthesia; GPE, general practitioners in emergency practice; GPG, general practitioners in non-emergency environment. In place-data are reported as counts [percentage of group total, 95% confidence interval]. P-value refers to comparisons among all groups*.

**Figure 2:**
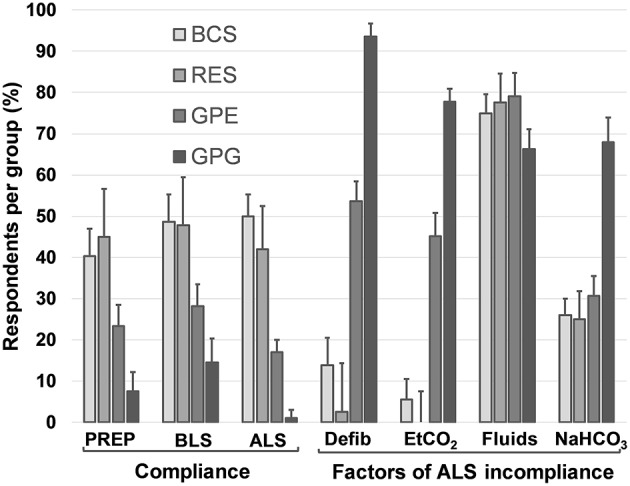
Preparedness, BLS and ALS compliance with RECOVER guidelines and the main factors leading to ALS incompliance. Data are reported as proportions of total respondents in each group. Error bars indicate 95% CI. PREP, preparedness; BCS, board-certified specialists in emergency and critical care and/or anesthesia; RES, residents in anesthesia or emergency and critical care; GPE, general practitioners in emergency practice; GPG, general practitioners in non-emergency environment; Defib, no defibrillator available; ETCO2, no routine use of capnography during CPR; Fluids, routine use of vascular expansion therapy during CPR; NaHCO_3_, sodium bicarbonate not readily available during CPR.

**Figure 3:**
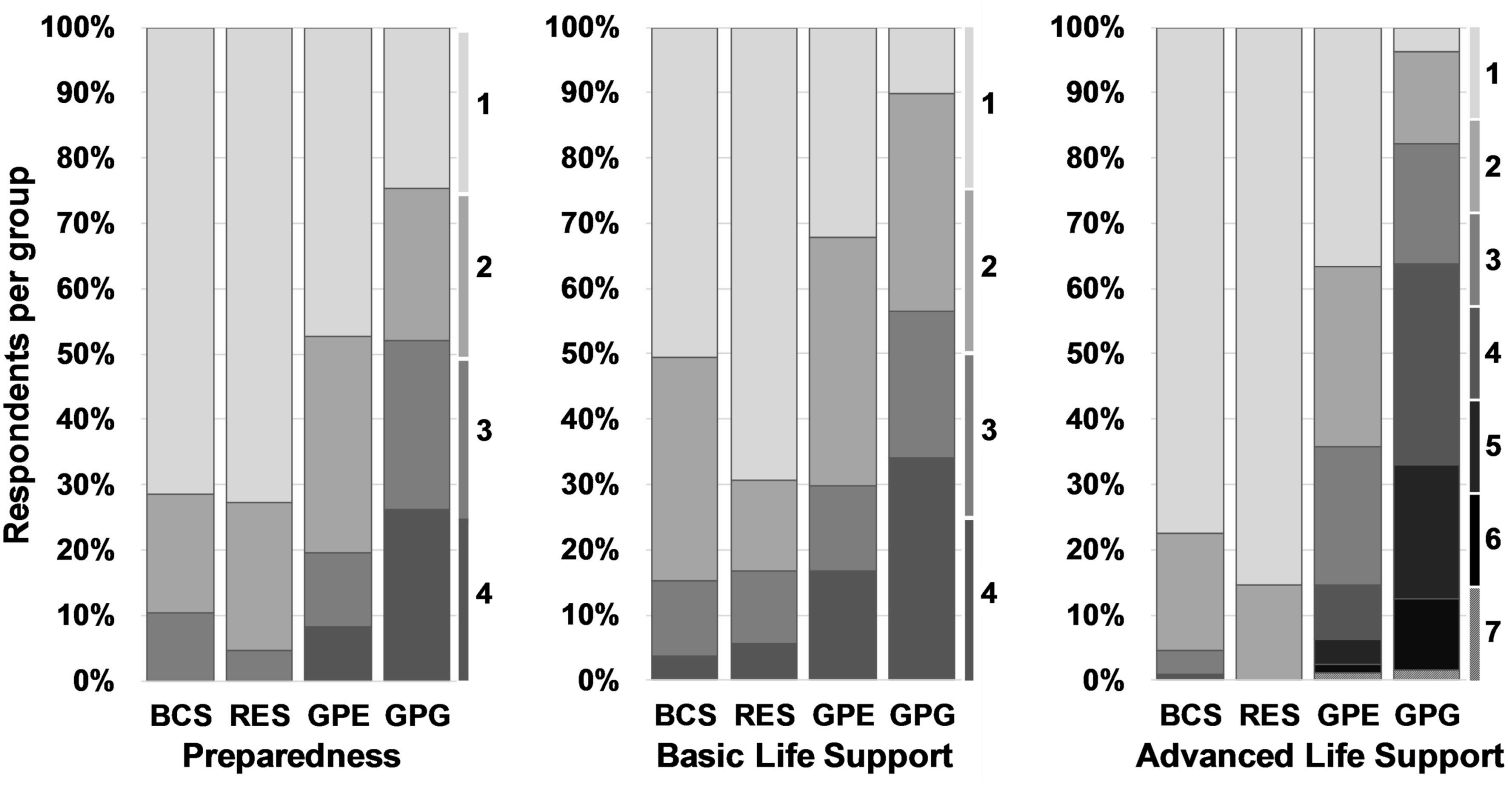
Number of incompliance factors in preparedness, BLS and ALS. Each column describes the group-wise proportion of respondents that disagrees in 1, 2, or more factors from complete RECOVER compliance. The numbers on the right to each domain's bar chart indicate the number of incompliant responses. BCS, board-certified specialists in emergency and critical care and/or anesthesia; RES, residents in anesthesia or emergency and critical care; GPE, general practitioners in emergency practice; GPG, general practitioners in non-emergency environment.

From the above responses, the proportion of those that responded in agreement with all preparedness items was similar in BCS (40% [95% CI: 34–47%]) and RES (45% [95% CI: 34–57%]), but significantly lower in GPE (23% [95% CI: 19–29%]) and further GPG (8% [95% CI: 5–12%]) (Figure 2). Non-compliant BCS and RES predominantly deviated in only one preparedness measure from the guidelines, while 53% of GPE and 75% of GPG disagreed in two or more points with the guidelines (Figure 3).

### Compliance With BLS Guidelines

**Figure 4:**
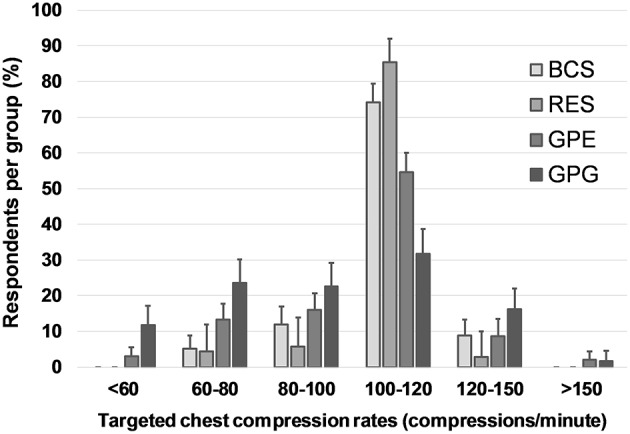
Targeted chest compression rates in dogs. Responses to the question: In dogs, with what frequency (compressions per minute) do you perform external chest compressions during CPR? Multiple choice question including seven categorical answers describing compression rate. Data are reported as proportions of total respondents in each group. Error bars indicate 95% CI. BCS, board-certified specialists in emergency and critical care and/or anesthesia; RES, residents in anesthesia or emergency and critical care; GPE, general practitioners in emergency practice; GPG, general practitioners in non-emergency environment.

**Figure 5:**
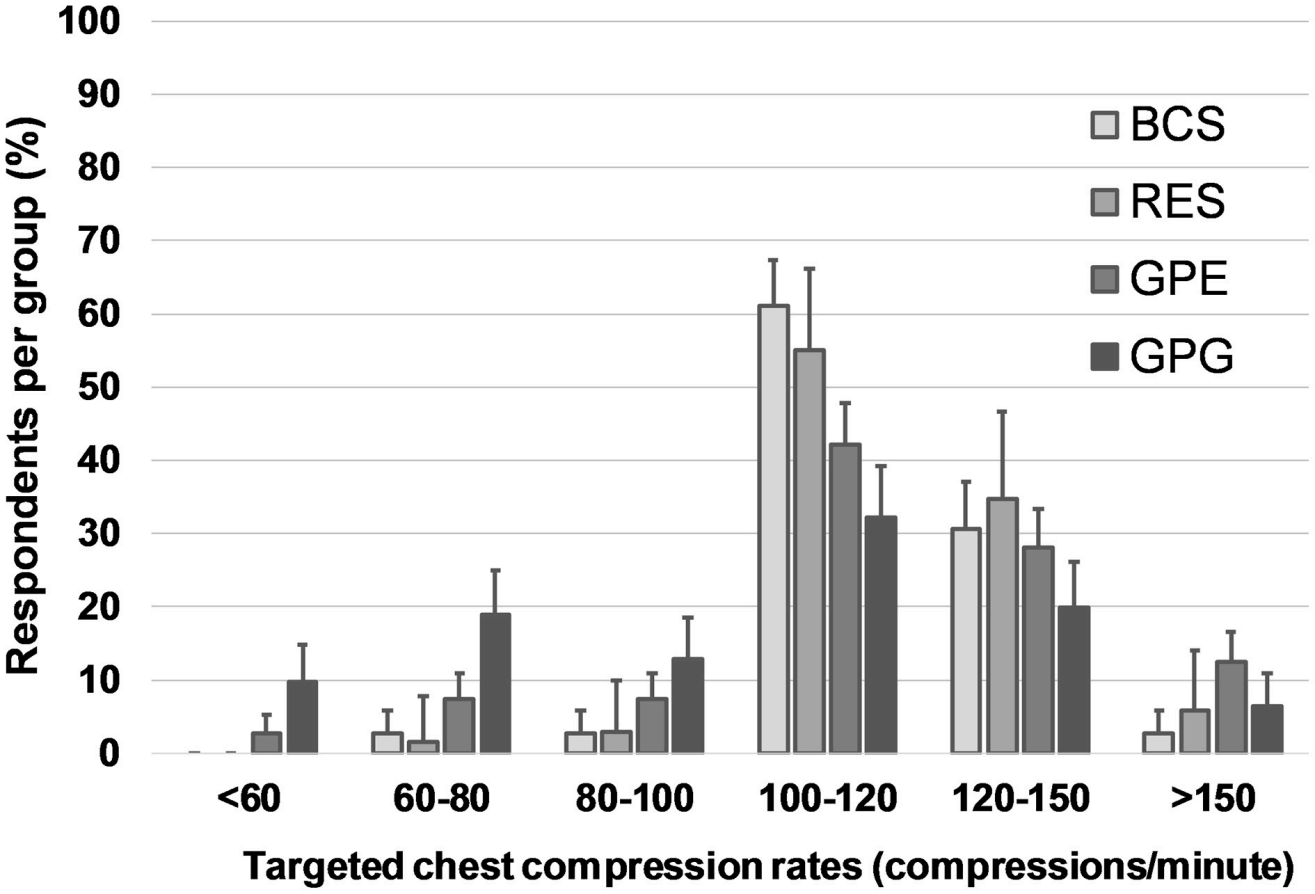
Targeted chest compression rates in cats. Responses to the question: In cats, with what frequency (compressions per minute) do you perform external chest compressions during CPR? Multiple choice question including seven categorical answers describing compression rate. Data are reported as proportions of total respondents in each group. Error bars indicate 95% CI. BCS, board-certified specialists in emergency and critical care and/or anesthesia; RES, residents in anesthesia or emergency and critical care; GPE, general practitioners in emergency practice; GPG, general practitioners in non-emergency environment.

In dogs, 86% (95% CI: 75–92%) of RES, 74% (95% CI: 68–80%) of BCS, 55% (95% CI: 49–60%) of GPE, and 32% (95% CI: 26–39%) of GPG targeted the currently recommended chest compression rate between 100 and 120 cpm. The majority of GPG (58% [95% CI: 51–65%]) selected rates < 100 cpm in dogs (Figure 4). Compared to dogs, respondents more frequently selected higher chest compression rates in cats, with 35% (95% CI: 32–39%) of all respondents aiming at more than 120 cpm, while only 10% (95% CI: 8–13%) choosing similar rates in dogs (*P* < 0.0001; Figure 5).

In both dogs (75%; 95% CI: 71–78%) and cats (73%; 95% CI: 70–76%), approximately three quarters of all respondents targeted a ventilation rate commensurate with current CPR guidelines (i.e., 6–15 breaths per minute). Of the remaining respondents, a higher percentage selected a ventilation rate that was higher than recommended (dogs: 18%; 95% CI: 16–21%; cats: 21%; 95% CI: 18–24%) compared with ventilation rates that were lower than recommended (dogs: 7%; 95% CI: 6–9%; cats: 6%; 95% CI: 5–8%) (*P* < 0.0001).

Taking the above findings together, nearly half of BCS (49%; 95% CI: 42–55%) and RES (48%; 95% CI: 36–59%) respondents' answers suggested BLS compliance, whereas less than a third of GPE (28%; 95% CI: 23–33%) and only 15% (95% CI: 10–20%) of GPG responded in adherence to BLS guidelines (Figure 2). The majority of BCS, RES and GPE, if not fully compliant, deviated only in one or two factors from the guidelines, while almost all GPG responded in disagreement with the guidelines in at least 2 points (Figure 3).

### Compliance With ALS Guidelines

The recommended monitoring modalities ECG and capnography (end-tidal carbon dioxide [ETCO_2_]) were widely available to BCS, RES, and GPE respondents, compared with 63% (95% CI: 56–70%) and 35% (95% CI: 29–42%) of GPG, respectively. Consequently, more than 95% of all BCS and RES respondents used ECG and capnography routinely, compared to 45% (ECG) and 23% (ETCO_2_) in GPG (Table 5). Similarly, the vast majority of BCS and RES respondents indicated that they had access to an electrical defibrillator, while this was the case in only 55% (95% CI: 50–61%) of GPE and 8% (95% CI: 5–12%) of GPG.

**Table 5:** Equipment available and used during ALS.

	**All groups**	**BCS**	**RES**	**GPE**	**GPG**	***P*-values**
ECG	635 [83, 80–85]	213 [99, 96–100]	68 [99, 92–100]	270 [90, 86–93]	84 [45, 38–52]	<0.0001
Capnograph	498 [65, 61–68]	207 [96, 92–98]	68 [99, 92–100]	181 [61, 55–66]	42 [23, 17–29]	<0.0001
Defibrillator	449 [58, 55–62]	201 [93, 89–96]	68 [99, 92–100]	166 [56, 50–61]	14 [8, 5–12]	<0.0001

*BCS, board-certified specialists in emergency and critical care and/or anesthesia; RES, residents in emergency and critical care and/or anesthesia; GPE, general practitioners in emergency practice; GPG, general practitioners in non-emergency environment. Data are reported as counts [percentage of group total, 95% confidence interval]. P-value refers to differences among all groups*.

Both atropine and epinephrine were widely available to all groups (Table 6). Large differences between groups existed regarding the availability of other drugs such as vasopressin and amiodarone (*P* < 0.0001) and a similar pattern emerged regarding sodium bicarbonate. Two thirds of both GPE (66%; 95% CI: 60–71%) and GPG (66%; 95% CI: 59–72%) indicated that they routinely integrated intravascular volume expansion therapy (e.g., crystalloid or colloid bolus or boluses) into their CPR strategy compared with 38% (95% CI: 31–44%) of BCS and 45% (95% CI: 34–57%) of RES. RECOVER does not recommend the routine use of IV fluids during CPR in euvolemic or hypervolemic dogs or cats (10).

**Table 6:** Drugs available during CPR attempts.

	**All groups**	**BCS**	**RES**	**GPE**	**GPG**	***P*-values**
Atropine	754 [98, 97–99]	216 [100, 98–100]	69 [100, 95–100]	295 [99, 97–100]	174 [94, 89–96]	<0.0001
Epinephrine	759 [99, 98–99]	216 [100, 98–100]	69 [100, 95–100]	298 [100, 98–100]	176 [95, 90–97]	<0.0001
Vasopressin	304 [39, 36–43]	130 [60, 54–67]	37 [54, 42–65]	120 [40, 35–46]	17 [9, 6–14]	<0.0001
Lidocaine	714 [93, 91–94]	209 [97, 94–99]	66 [96, 88–99]	288 [96, 94–98]	151 [81, 75–86]	<0.0001
Amiodarone	205 [27, 24–30]	83 [38, 32–45]	42 [61, 49–72]	64 [21, 17–26]	16 [9, 5–14]	<0.0001
Sodium bicarbonate	531 [69, 66–72]	188 [87, 82–91]	59 [86, 75–92]	223 [75, 69–79]	61 [33, 26–40]	<0.0001

*BCS, board-certified specialists in emergency and critical care and/or anesthesia; RES, residents in emergency and critical care and/or anesthesia; GPE, general practitioners in emergency practice; GPG, general practitioners in non-emergency environment. Data are reported as counts [percentage of group total, 95% confidence interval]. P-value refers to differences among all groups*.

Fifty percent of BCS (95% CI: 43–57%) and 42% (95% CI: 31–54%) of RES complied with ALS guidelines compared with 17% (95% CI: 13–22%) of GPE and 1% (95% CI: 0–4%) of GPG (Figure 2). Lack of access to a defibrillator and not routinely using capnography during CPR were common sources of ALS non-compliance in GPE and GPG, but not in BCS or RES. Routine use of intravascular volume expansion therapy was a factor of ALS non-compliance present in more than 60% of the respondents in all groups and the predominant cause for incompliance in BCS and RES. Limiting ALS compliance criteria to those recommendations with higher levels of evidence I or IIa (i.e., epinephrine or vasopressin, atropine, ECG, ETCO_2_, and defibrillator), 89% (95% CI: 85–93%) of BCS and 96% (95% CI: 88–99%) of RES complied with ALS guidelines compared with 43% (95% CI: 38–49%) of GPE and 3% (95% CI: 2–7%) of GPG. While disagreement with one ALS guideline explained most of the incompliance in BCS and RES, a majority of GPE respondents were incompliant due to at least two recommendations and GPG respondents deviated in several points from the guidelines (Figure 3).

### RECOVER Awareness and CPR Compliance

A total of 576 respondents (76%; 95% CI: 72–78%) indicated that they had heard of the 2012 RECOVER CPR guidelines, which included all BCS (100%; 95% CI: 98–100%) and RES (100%; 95% CI: 95–100%), and 77% (95% CI: 72–81%) of GPE but only 35% (95% CI: 28–42%) of GPG. Of those that were aware of this resource, most (*n* = 542; 93% [95% CI: 91–95%]) consulted the RECOVER guidelines, including 100% (95% CI: 97–100%) of BCS, 97% (95% CI: 90–99%) of RES, 91% (95% CI: 87–94%) of GPE, and 77% (95% CI: 65–85%) of GPG.

All professional groups taken together, respondents that expressed awareness of the RECOVER guidelines were significantly more compliant in preparedness (32%; 95% CI: 29–36%), BLS (40%; 95% CI: 36–44%), and ALS (32%; 95% CI: 29–36%) compared to those not aware of the guidelines (preparedness: 7%; 95% CI: 4–12%; BLS: 8%; 95% CI: 5–13%; ALS: 1%; 95% CI: 0–4%). After adjusting for the effect of respondent age, gender, region of work and professional group the odds of a respondent aware of the RECOVER guidelines being preparedness-compliant was 2.4 (95% CI: 1.2–4.8, *P* = 0.0086) times, being BLS-compliant was 4.5 (95% CI: 2.4–9.1, *P* < 0.0001) times, and being ALS-compliant was 7.8 (95% CI: 2.3–49.1, *P* < 0.0001) times the odds of a non-aware respondent being compliant in these domains.

## Discussion

Survival from CPA is not possible without CPR and evidence suggests that survival rates from cardiac arrest, while very low, are not static, but can be improved by high-quality CPR delivery (18, 19). This includes early recognition and response to CPA, and delivery of effective BLS, ALS and post-cardiac arrest care (7, 20, 21). According to the “chain of survival” paradigm, a single inadequately executed element of the chain will compromise the outcome potential of a patient (20). Current best practice CPR in both human and veterinary medicine is derived from evidence- and consensus-based treatment recommendations and CPR is generally considered excellent when it complies with these guidelines (10, 22). Implementation of CPR guidelines in people led to improved outcomes (23–25). To improve health care system-wide outcomes from CPA, leaders in the human resuscitation community developed a formula for survival that includes the domains of medical science, educational efficiency and local implementation. This formula proposes that survival depends on consistently excellent fulfillment of domain objectives: failure of any of the domains (e.g., poor medical science) will invariably lead to failure of the entire system (i.e., poor survival rate), no matter how good the others (e.g., educational efficiency and local implementation) are executed (26).

Our study examined the self-reported adherence of clinically active veterinarians to a subset of the 101 RECOVER CPR guidelines published 5 years prior to this survey (10). The main finding of our study is that the respondents' veterinary CPR practice does often not meet guidelines. The level of disagreement is likely a conservative estimate as not all RECOVER recommendations were queried in the survey. For example, in the domain of BLS, important characteristics such as animal position, chest compression point, chest compression depth, recoil and minimization of pauses were not investigated (10). In a previous CPR survey conducted in 2008, large variation in self-reported CPR practices suggests a qualitatively similar disagreement with guidelines (8). Of note, a common, authoritative standard such as the RECOVER guidelines were not yet available at that time. In human medicine, deviations from recommended CPR techniques were found to be highly prevalent and more recently, analysis of defibrillator recordings of various chest compression quality metrics provided good insight into actual clinical CPR performance (23, 27–29). One study analyzing data from an international registry with 19,568 out-of-hospital CPA cases with full CPR quality data available, found that only 15% of patients were treated with guideline-compliant CPR (23). Sutton et al. described the adherence to recommended chest compression rates in an in-hospital pediatric critical care setting; among the 164 patients in the study, compression rates were delivered according to guidelines only 32.6% of the time (27). These studies did not report the knowledge of CPR guidelines of the medical professionals. Thus, it remains unclear whether the rescuers in these studies performed better or worse than their theoretical knowledge would suggest. However, research including human CPR-certified EMS personnel, medical students, and emergency room personnel suggests that better theoretical knowledge of BLS guidelines is positively associated with guideline-compliant CPR performance on manikins (30, 31). In one of these studies, 86% of those indicating a guideline-compliant compression rate in a survey performed BLS with the recommended rate, whereas only 46% of survey responders indicating a non-compliant compression rate performed BLS with the recommended rate (31). We could not identify any published evidence to support the notion that rescuers are more likely to perform CPR in agreement with guidelines than their theoretical knowledge of the guidelines would suggest. It is therefore plausible to assume that the lack of adherence with guidelines that we found in our study will also be present to the same or a more pronounced extent in actual clinical practice. It would nevertheless be desirable to determine the actual compliance in a simulation or, more importantly, a clinical case management context.

Not surprisingly, compared to BCS and RES, GPE and GPG were less compliant overall and when non-compliant the level of deviation from guidelines was greater. In BCS and RES, non-adherence to the recommendations in any domain was due to a single factor in the majority of respondents. Accordingly, the adoption of a few changes in CPR practice including shortened refresher training intervals, uniform administration of chest compressions at the recommended rate in both dogs and cats, restricting resuscitative fluid therapy during CPR to hypovolemic patients and having sodium bicarbonate readily available for use during CPR would largely abolish incompliances in BCS and RES. Since not all RECOVER guidelines were evaluated in our study other cognitive CPR elements (e.g., compression depth) may require remediation. This study suggests that for BCS and RES respondents, further investments in the infrastructural environment may not be necessary as almost all had access to crash cart, ECG, capnography and defibrillator, cognitive aids, and resuscitation team sizes of 4 or more.

In contrast, GPE and especially GPG, more commonly disagreed with recommended CPR techniques (e.g., chest compression rates) and were also challenged by limitations in resuscitation infrastructure. As for BCS and RES, more frequent training sessions would likely be effective in resolving most knowledge gaps and disagreements with guidelines as well as in improving CPR psychomotor skills, with more frequent training leading to better performance (32, 33). A RECOVER guidelines-directed hospital wide CPR training program in a veterinary emergency clinic in Japan (i.e., falling into the category of GPE) was associated with an increase in return of spontaneous circulation (ROSC) rates in dogs from 20 to 59% (*P* < 0.01) and an increase, albeit not statistically significant, in survival to discharge from 0 to 9% (34). However, even competent GPE and GPG rescuers may not be able to apply CPR according to guidelines, as our findings suggest that capnography and defibrillators are commonly not available and team performance may be constrained by small team size. Recent systematic reviews and meta-analyses in human medicine concluded that higher ETCO_2_ values are associated with ROSC and that ETCO_2_ monitoring during CPR may have some prognostic value (35–37). Similarly, veterinary data in dogs and cats suggests that low ETCO_2_ values during CPR (i.e., <15 mm Hg in dogs and <20 mm Hg in cats) are associated with an increased risk of failure to achieve ROSC (3). This finding was further corroborated in a recent prospective observational study in dogs and cats undergoing CPR that found a positive association between initial, peak and mean ETCO_2_ values as well as ETCO_2_ variation over time with ROSC (38). Regarding the availability and importance of defibrillators, considering that the intra-arrest occurrence rate of ventricular fibrillation has been reported to be as high as 34% in dogs and 21% in cats, the acquisition of a defibrillator, could be meaningful in the GPG setting, particularly in clinics performing frequent anesthetic procedures (4, 39).

Delivering guideline-compliant CPR requires three components: awareness of the guidelines, consultation with them and delivery of CPR according to them. All BCS and RES and 77% of GPE respondents indicated that they had heard of the RECOVER guidelines, however only slightly more than a third of GPG respondents had. Those respondents that had heard of RECOVER were, independent of age, gender, region of practice or professional group, markedly more likely to answer BLS and ALS questions in compliance with the guidelines compared to those that had not. This indicates that awareness of the guidelines may have influenced the self-reported clinical practice in CPR in a positive way. Most of those that were aware of the guidelines did in fact consult this resource. However, despite this awareness and consultation, only half of the respondents in the BCS and RES groups indicated BLS and ALS techniques in compliance with the recommendations. Even-though these findings result from knowledge assessment only, evidence in human medicine suggests that rescuers with higher theoretical knowledge of CPR guidelines are more likely to deliver guideline-compliant CPR (30, 31). Studies in human medicine have further shown that even highly trained health care providers do not consistently adhere to published CPR guidelines (40). Inadequate chest compression rate and depth, as well as hyperventilation, were commonly identified deficiencies in CPR practices during out-of-hospital and in-hospital cardiac arrest (40–42).

The limitations of this survey-based study require careful consideration. While advantageous in terms of time, cost and workflow efficiency, internet-based surveys are not without disadvantages and this study is no exception (43–45). Several studies surveying medical professionals have demonstrated significantly lower response rates associated with the digital approach compared with postal-based surveys (45–47). This study's response rate was indeed low despite a relatively large sample size. The actual response rate is difficult to determine as a proportion of the invited recipients would not have fulfilled the stated participation inclusion criteria and the number of respondents participating through links shared via social media outlets cannot be quantified. In addition, not all relevant organization (e.g., the Association of Veterinary Anaesthetists) were invited to the survey, which could have influenced the profile of BCS respondents and thus the content of the analyzed data set. Another limitation of this study is self-selection bias: recipients with a greater interest in CPR likely responded more readily than recipients with a lesser interest, leading to a possible overestimation of compliance compared to the entire population. In addition, it is possible that more than one respondent from the same veterinary hospital participated in the survey. While it is uncertain how this may have influenced the findings of this study, data presented in Table 1 offers insight into the practice setting of the four groups. To minimize the risk of further bias there was no mention of the RECOVER initiative in the email invitations or survey introduction. Additionally, it is important to recognize that a likely discrepancy between respondents' self-reported CPR practices and their actual CPR technique exists, so that our data are not synonymous with actual CPR performance in clinical practice (48). However, it is unlikely that actual CPR performance is in higher agreement with guidelines than that which is self-reported (30, 31). Despite the low response rate, respondent demographics appear representative of the respective source population at least in as far as the North American veterinary population goes. Age distribution of respondents was consistent with expectations (e.g., BCS oldest, RES youngest) and gender distribution was reasonably in agreement with recent reports of the age and gender composition of US veterinary professionals (49). Board certified specialists were older than RES as to be expected. Responses were consistent with professional groups across all questions. We therefore believe that the findings of this study are an acceptable estimate of guideline compliance in clinical practice of CPR in dogs and cats. Until small animal CPR registry data are available that also incorporate collection of CPR quality variables, such as chest compression rate, depth, recoil, and chest compression fraction, our findings are the best approximation to the adherence of CPR providers to current guidelines.

In conclusion, awareness of RECOVER guidelines is high in specialists and residents, but incomplete among general practitioners. Awareness of these guidelines positively influenced compliance, but CPR practices continue to be variable and commonly not in agreement with current treatment recommendations. Widely accessible, cost- and time-effective educational and implementation strategies are required to broadly improve knowledge and application of best practices in small animal CPR. Such programs need to take into consideration the different professional environments and infrastructural constraints in which CPR is applied.

## Data Availability

The datasets generated for this study are available on request to the corresponding author.

## Ethics Statement

The survey procedures and questionnaire were reviewed and approved by the Veterinary and Agricultural Sciences Human Ethics Advisory Group of the University of Melbourne as a Minimal Risk Project. The consent was implied when participating to the survey. Project title: Clinical practice of small animal cardiopulmonary resuscitation after publication of standardized guidelines: an internet based survey. Ethics ID: 1546061

## Author Contributions

ÍG designed the survey questionnaire, interpreted data, and wrote the manuscript. DF helped design the survey questionnaire and critically revised the manuscript. MS analyzed and interpreted the data and critically revised the manuscript. MB designed the study questionnaire, conducted the survey, analyzed and interpreted data, and critically revised the manuscript. All authors read and approved the final manuscript.

### Conflict of Interest Statement

MB and DF are co-chairs of the RECOVER initiative that released the RECOVER guidelines. The remaining authors declare that the research was conducted in the absence of any commercial or financial relationships that could be construed as a potential conflict of interest.

## Footnotes

^1^Microsoft Excel, Office 365, Microsoft, Redmond, WA.

^2^JMP, Version 12, SAS Institute Inc., Cary, NC 1989–2019.

## Abbreviations

ALS: advanced life support
BCS: board certified specialists
BLS: basic life support
CPA: cardiopulmonary arres
CPR: cardiopulmonary resuscitation
ETCO_2_: end tidal CO_2_
GPE: general practitioners working in emergency practices
GPG: general practitioners working in general practices
RECOVER: Reassessment Campaign on Veterinary Resuscitation
RES: residents
ROSC: return of spontaneous circulation.
