# Supplementary material for: The Compliance of Current Small Animal CPR Practice With RECOVER Guidelines: An Internet-Based Survey
Source: Front Vet Sci. 2019 Jun 11;6:181. doi: 10.3389/fvets.2019.00181 (PMC6581025; doi:10.3389/fvets.2019.00181)
Supplement: Supplementary file 1 [file Data_Sheet_1.pdf]

Survey: The clinical practice of small animal CPR

Thank you for contributing your time and experience to this project!

This survey is research, and by responding to the survey you are participating in a research project. Your participation is entirely voluntary. No personal identifying data will be used for this research and your identity will be protected. Please do not hesitate to contact us with any questions or concerns (manuel.boller@unimelb.edu.au).

IMPORTANT: If you need to leave the survey before completing it: click on the “select and return later” button at the bottom of each page. You will be given a return code to log in to your survey in the future. Please remember the code!

Sincerely,

Manuel, Dan, and Íde

Manuel Boller, University of Melbourne

Dan Fletcher, Cornell University

Íde Gillespie, University of Melbourne

Study contact:

Manuel Boller, Dr. med. vet., MTR, DACVECC

University of Melbourne

250 Princes Highway

Werribee, VIC 3013, Australia

Phone: +61 3 8001 2508

Email: manuel.boller@unimelb.edu.au

Eligibility to survey

Do you currently provide any clinical veterinary care to small animals (dogs and/or cats)?

◯ Yes
◯ No

Section 1: Tell us about yourself

What is your gender?

- ◯ Female
- ◯ Male
- ◯ Other
- ◯ Prefer not to answer

What is your age?

____
(Age in years)

In what country or world region are you currently practicing veterinary medicine?

- ◯ Africa
- ◯ Asia
- ◯ Australia or New Zealand
- ◯ Europe
- ◯ Canada
- ◯ Latin America
- ◯ United States of America
- ◯ I am not in clinical practice
- ◯ Other

Which best describes your current professional status?

- ◯ General practitioner (including unspecialized ER vet)
- ◯ Board certified specialist
- ◯ Veterinary student
- ◯ Intern
- ◯ Resident
- ◯ Veterinary nurse or technician
- ◯ Not currently in clinical practice or studying veterinary medicine
- ◯ Other

What other?

____

What year did you graduate from veterinary school?

- ◯ 2017
- ◯ 2016
- ◯ 2015
- ◯ 2014
- ◯ 2013
- ◯ 2012
- ◯ 2011
- ◯ 2010
- ◯ 2009
- ◯ 2008
- ◯ 2007
- ◯ 2006
- ◯ 2005
- ◯ 2004
- ◯ 2003
- ◯ 2002
- ◯ 2001
- ◯ 2000
- ◯ 1999
- ◯ 1998
- ◯ 1997
- ◯ 1996
- ◯ 1995
- ◯ 1994
- ◯ 1993
- ◯ 1992
- ◯ 1991
- ◯ 1990
- ◯ 1989
- ◯ 1988
- ◯ 1987
- ◯ 1986
- ◯ 1985
- ◯ 1984
- ◯ 1983
- ◯ 1982
- ◯ 1981
- ◯ 1980
- ◯ 1979
- ◯ 1978
- ◯ 1977
- ◯ 1976
- ◯ 1975
- ◯ 1974
- ◯ 1973
- ◯ 1972
- ◯ 1971
- ◯ 1970
- ◯ 1969
- ◯ 1968
- ◯ 1967
- ◯ 1966
- ◯ 1965
- ◯ 1964
- ◯ 1963
- ◯ 1962
- ◯ 1961
- ◯ 1960
- ◯ 1959
- ◯ 1958
- ◯ 1957
- ◯ 1956
- ◯ 1955
- ◯ 1954
- ◯ 1953
- ◯ 1952
- ◯ 1951
- ◯ 1950

Where are you fulfilling your internship requirement?

- ◯ University
- ◯ Private practice

What is the internship in which you are enrolled?

- ◯ Small animal, rotating
- ◯ Small animal emergency medicine only
- ◯ Large animal
- ◯ Other

Please specify ______________________________

In which year of your residency are you?

- ◯ First
- ◯ Second
- ◯ Third
- ◯ Fourth
- ◯ Fifth
- ◯ Sixth or more

Where are you fulfilling your residency requirement?

- ◯ University
- ◯ Private practice

What is the residency in which you are enrolled?

- ◯ Anesthesia
- ◯ Animal Behavior
- ◯ Cardiology
- ◯ Dentistry
- ◯ Dermatology
- ◯ Emergency and Critical Care, large animal
- ◯ Emergency and Critical Care, small animal
- ◯ Internal Medicine, large animal
- ◯ Internal Medicine, small animal
- ◯ Laboratory Animal Medicine
- ◯ Neurology
- ◯ Nutrition
- ◯ Oncology
- ◯ Ophthalmology
- ◯ Pathology or Clinical Pathology
- ◯ Pharmacology
- ◯ Radiology
- ◯ Shelter Medicine
- ◯ Surgery, large animal
- ◯ Surgery, small animal
- ◯ Theriogenology/Reproductive Medicine
- ◯ Zoo & Wildlife Medicine
- ◯ Other

Please specify: ______________________________

By which veterinary specialty organization are you board certified?

Check all that apply.

- ☐ ABVP
- ☐ ACVAA
- ☐ ACVECC
- ☐ ACVIM
- ☐ ACVP
- ☐ ACVS
- ☐ ANZCVS-ECC
- ☐ ECVAA
- ☐ ECVECC
- ☐ ECVIM
- ☐ ECVS
- ☐ Other

Please specify: ______________________________

What year did you fulfill your residency requirements?

◯ 2017
◯ 2016
◯ 2015
◯ 2014
◯ 2013
◯ 2012
◯ 2011
◯ 2010
◯ 2009
◯ 2008
◯ 2007
◯ 2006
◯ 2005
◯ 2004
◯ 2003
◯ 2002
◯ 2001
◯ 2000
◯ 1999
◯ 1998
◯ 1997
◯ 1996
◯ 1995
◯ 1994
◯ 1993
◯ 1992
◯ 1991
◯ 1990
◯ 1989
◯ 1988
◯ 1987
◯ 1986
◯ 1985
◯ 1984
◯ 1983
◯ 1982
◯ 1981
◯ 1980
◯ 1979
◯ 1978
◯ 1977
◯ 1976
◯ 1975
◯ 1974
◯ 1973
◯ 1972
◯ 1971
◯ 1970
◯ 1969
◯ 1968
◯ 1967
◯ 1966
◯ 1965
◯ 1964
◯ 1963
◯ 1962
◯ 1961
◯ 1960
◯ 1959
◯ 1958
◯ 1957
◯ 1956
◯ 1955
◯ 1954
◯ 1953
◯ 1952
◯ 1951
◯ 1950

(If you completed several residencies, indicate the completion year for the most recent

residency.)

Where did you fulfill your residency requirements?	◯ University<br>◯ Private practice<br>(If you completed several residencies, answer this question for the most recent residency.)

What year did you graduate from veterinary nursing/technician school?

- ◯ 2017
- ◯ 2016
- ◯ 2015
- ◯ 2014
- ◯ 2013
- ◯ 2012
- ◯ 2011
- ◯ 2010
- ◯ 2009
- ◯ 2008
- ◯ 2007
- ◯ 2006
- ◯ 2005
- ◯ 2004
- ◯ 2003
- ◯ 2002
- ◯ 2001
- ◯ 2000
- ◯ 1999
- ◯ 1998
- ◯ 1997
- ◯ 1996
- ◯ 1995
- ◯ 1994
- ◯ 1993
- ◯ 1992
- ◯ 1991
- ◯ 1990
- ◯ 1989
- ◯ 1988
- ◯ 1987
- ◯ 1986
- ◯ 1985
- ◯ 1984
- ◯ 1983
- ◯ 1982
- ◯ 1981
- ◯ 1980
- ◯ 1979
- ◯ 1978
- ◯ 1977
- ◯ 1976
- ◯ 1975
- ◯ 1974
- ◯ 1973
- ◯ 1972
- ◯ 1971
- ◯ 1970
- ◯ 1969
- ◯ 1968
- ◯ 1967
- ◯ 1966
- ◯ 1965
- ◯ 1964
- ◯ 1963
- ◯ 1962
- ◯ 1961
- ◯ 1960
- ◯ 1959
- ◯ 1958
- ◯ 1957
- ◯ 1956
- ◯ 1955
- ◯ 1954
- ◯ 1953
- ◯ 1952
- ◯ 1951
- ◯ 1950

Do you hold specialty certification in veterinary ECC (e.g., VTS[ECC])?

◯ Yes
◯ No

Section 2: Describe you working environment

How many veterinary clinicians work in your current place of employment?

- ◯ 1
- ◯ 2
- ◯ 3
- ◯ 4
- ◯ 5
- ◯ 6
- ◯ 7
- ◯ 8
- ◯ 9
- ◯ 10
- ◯ 11
- ◯ 12
- ◯ 13
- ◯ 14
- ◯ 15
- ◯ 16
- ◯ 17
- ◯ 18
- ◯ 19
- ◯ 20
- ◯ 21
- ◯ 22
- ◯ 23
- ◯ 24
- ◯ 25
- ◯ 26
- ◯ 27
- ◯ 28
- ◯ 29
- ◯ 30
- ◯ 31
- ◯ 32
- ◯ 33
- ◯ 34
- ◯ 35
- ◯ 36
- ◯ 37
- ◯ 38
- ◯ 39
- ◯ 40
- ◯ 41
- ◯ 42
- ◯ 43
- ◯ 44
- ◯ 45
- ◯ 46
- ◯ 47
- ◯ 48
- ◯ 49
- ◯ 50
- ◯ 51
- ◯ 52
- ◯ 53
- ◯ 54
- ◯ 55
- ◯ 56
- ◯ 57
- ◯ 58
- ◯ 59
- ◯ 60
- ◯ 61
- ◯ 62
- ◯ 63
- ◯ 64
- ◯ 65
- ◯ 66
- ◯ 67
- ◯ 68
- ◯ 69
- ◯ 70

◯ 71
◯ 72
◯ 73
◯ 74
◯ 75
◯ 76
◯ 77
◯ 78
◯ 79
◯ 80
◯ 81
◯ 82
◯ 83
◯ 84
◯ 85
◯ 86
◯ 87
◯ 88
◯ 89
◯ 90
◯ 91
◯ 92
◯ 93
◯ 94
◯ 95
◯ 96
◯ 97
◯ 98
◯ 99
◯ 100
◯ >100

What is the daily average number of dogs and cats that you personally attend to at your clinic?

◯ None
◯ 1 to 5
◯ 6 to 10
◯ 11 to 20
◯ More than 20

Which of the following best describes the emergency service your clinic provides?

◯ Emergency service during regular business hours only
◯ Emergency service during regular business hours and on call service after hours
◯ Emergency clinic or service open after regular business hours only
◯ Emergency clinic or service open 24hrs and able to hospitalize patients
◯ Emergency/critical care center with at least one board-certified emergency clinician
◯ Other

Please specify:

____

Which of the following best describes your workplace?

◯ Private practice (general or specialty) with no interns or residents
◯ Private practice with interns
◯ Private practice with residents
◯ Private practice with interns and residents
◯ University practice

Which of the following best describes your clinic's patient distribution?

- ◯ Small animals (including exotics) exclusively
- ◯ Cats only
- ◯ Dogs only
- ◯ Mixed, with more than 50% small animals
- ◯ Mixed, with less than 50% small animals
- ◯ Other

Please specify: __________________________________

Section 3: Describe your patients

For which species are you personally providing medical care?

- ◯ Small animals including avian and exotics
- ◯ Dogs only
- ◯ Cats only
- ◯ Dogs and cats only
- ◯ Small and large animals
- ◯ Other

Please specify:

____

Approximately what percentage of cats and dogs that you personally see, present as emergencies?

- ◯ None
- ◯ 1% to 10%
- ◯ 11% to 25%
- ◯ 26% to 50%
- ◯ 51% to 75%
- ◯ 76% to 99%
- ◯ 100%

Section 4: Describe your CPR experience

How long has it been since you last participated in some form of veterinary CPR training?

◯ Within the last 6 months
◯ Between 6 months and 1 year ago
◯ Between 1 and 3 years ago
◯ More than 3 years ago
◯ Never did any CPR training

How many times on average per year are you personally involved in performing CPR?

◯ Never
◯ 1 time
◯ 2 to 5 times
◯ 6 to 10 times
◯ 11 to 20 times
◯ More than 20 times

On average, how many people make up the resuscitation team during CPR in your practice?

◯ 1 or 2
◯ 3
◯ 4
◯ 5
◯ More than 5

Section 5: CPR preparedness

Which of the following preparedness measures for CPR are in place in your practice?

Check all that apply.

- ☐ Continuing in-house education in veterinary CPR
- ☐ Regular training drills for staff likely to be involved in CPR
- ☐ Regularly maintained 'crash cart' or 'crash station'
- ☐ Emergency drug dosing chart displayed
- ☐ CPR algorithm displayed
- ☐ Specific CPR record sheet to document the resuscitation attempt
- ☐ None of the above

How important do you think these preparedness measures are?

Continuing in-house education in veterinary CPR

○ 1 (=no importance) ○ 2 ○ 3 ○ 4 ○ 5 ○ 6 ○ 7 ○ 8 ○ 9 ○ 10 (=absolutely necessary)

Regular training drills for staff likely to be involved in CPR

○ 1 (=no importance) ○ 2 ○ 3 ○ 4 ○ 5 ○ 6 ○ 7 ○ 8 ○ 9 ○ 10 (=absolutely necessary)

Regularly maintained 'crash cart' or 'crash station'

○ 1 (=no importance) ○ 2 ○ 3 ○ 4 ○ 5 ○ 6 ○ 7 ○ 8 ○ 9 ○ 10 (=absolutely necessary)

Emergency drug dosing chart displayed

○ 1 (=no importance) ○ 2 ○ 3 ○ 4 ○ 5 ○ 6 ○ 7 ○ 8 ○ 9 ○ 10 (=absolutely necessary)

CPR algorithm displayed

○ 1 (=no importance) ○ 2 ○ 3 ○ 4 ○ 5 ○ 6 ○ 7 ○ 8 ○ 9 ○ 10 (=absolutely necessary)

Specific CPR record sheet to document the resuscitation attempt

○ 1 (=no importance) ○ 2 ○ 3 ○ 4 ○ 5 ○ 6 ○ 7 ○ 8 ○ 9 ○ 10 (=absolutely necessary)

Section 6: Generating blood flow

Which of the following techniques have you employed during CPR?

Check all that apply.

- ☐ Closed-chest compressions
- ☐ Open-chest CPR
- ☐ Aortic cross clamping
- ☐ Interposed (alternating) abdominal compressions
- ☐ Impedance threshold device (ITD)
- ☐ None of the above

In DOGS, with what frequency (compressions per minute) do you perform external chest compressions during CPR?

◯ Less than 60 ◯ 60 to 80 ◯ 80 to 100 ◯ 100 to 120 ◯ 120 to 150 ◯ 150 to 200
◯ More than 200

In CATS, with what frequency (compressions per minute) do you perform external chest compressions during CPR?

◯ Less than 60 ◯ 60 to 80 ◯ 80 to 100 ◯ 100 to 120 ◯ 120 to 150 ◯ 150 to 200
◯ More than 200

Section 7: Ventilation

By which means have you supported breathing during CPR in dogs or cats?

Check all that apply.

- ☐ Mouth-to-snout ventilation
- ☐ Mask and ambu-bag
- ☐ Endotracheal intubation
- ☐ Emergency tracheostomy
- ☐ Supraglottic airway
- ☐ Jen Chung (GV 26) acupuncture point stimulation
- ☐ Other

Please specify:

____

What gas do you routinely use for delivering breaths during CPR?

Check all that apply.

- ☐ Supplemental oxygen
- ☐ Room air only
- ☐ Exhaled breath

In DOGS, what is the ventilation rate (breaths per minute) you are generally targeting during CPR?

- ◯ 1 to 5
- ◯ 6 to 15
- ◯ 16 to 30
- ◯ 31 to 45
- ◯ 46 to 60
- ◯ To match compression rate
- ◯ As many as possible

In CATS, what is the ventilation rate (breaths per minute) you are generally targeting during CPR?

- ◯ 1 to 5
- ◯ 6 to 15
- ◯ 16 to 30
- ◯ 31 to 45
- ◯ 46 to 60
- ◯ To match compression rate
- ◯ As many as possible

How do you deliver breaths in relation to chest compressions in the intubated animal?

- ◯ One breath during each compression
- ◯ One breath between each compression
- ◯ Breaths delivered during compressions to achieve target respiratory rate
- ◯ Breaths delivered between compressions to achieve target respiratory rate
- ◯ Breaths given independent of compressions to achieve target respiratory rate

Section 8: CPR monitoring

Which of the following monitoring tools or techniques do you have available at your clinic for use during CPR?

Check all that apply.

- ☐ ECG
- ☐ Capnograph
- ☐ Pulse oximeter
- ☐ Oscillometric BP measurement
- ☐ Doppler BP measurement
- ☐ Ultrasound
- ☐ Stethoscope
- ☐ None of the above

Which do you routinely use during CPR?

Check all that apply.

- ☐ ECG
- ☐ Capnograph
- ☐ Pulse oximeter
- ☐ Oscillometric BP measurement
- ☐ Doppler BP measurement
- ☐ Ultrasound
- ☐ Direct pulse palpation
- ☐ Palpation of apex beat
- ☐ Stethoscope
- ☐ Mucous membrane color and CRT

How important do you think these monitoring techniques are during CPR?

ECG (electrocardiogram)

◯ 1 (=no importance) ◯ 2 ◯ 3 ◯ 4 ◯ 5 ◯ 6 ◯ 7 ◯ 8 ◯ 9 ◯ 10 (=absolutely necessary)

Capnograph

◯ 1 (=no importance) ◯ 2 ◯ 3 ◯ 4 ◯ 5 ◯ 6 ◯ 7 ◯ 8 ◯ 9 ◯ 10 (=absolutely necessary)

Pulse oximeter

◯ 1 (=no importance) ◯ 2 ◯ 3 ◯ 4 ◯ 5 ◯ 6 ◯ 7 ◯ 8 ◯ 9 ◯ 10 (=absolutely necessary)

Oscillometric BP measurement

◯ 1 (=no importance) ◯ 2 ◯ 3 ◯ 4 ◯ 5 ◯ 6 ◯ 7 ◯ 8 ◯ 9 ◯ 10 (=absolutely necessary)

Doppler BP measurement

◯ 1 (=no importance) ◯ 2 ◯ 3 ◯ 4 ◯ 5 ◯ 6 ◯ 7 ◯ 8 ◯ 9 ◯ 10 (=absolutely necessary)

Ultrasound

◯ 1 (=no importance) ◯ 2 ◯ 3 ◯ 4 ◯ 5 ◯ 6 ◯ 7 ◯ 8 ◯ 9 ◯ 10 (=absolutely necessary)

Direct pulse palpation

◯ 1 (=no importance) ◯ 2 ◯ 3 ◯ 4 ◯ 5 ◯ 6 ◯ 7 ◯ 8 ◯ 9 ◯ 10 (=absolutely necessary)

Palpation of apex beat

◯ 1 (=no importance) ◯ 2 ◯ 3 ◯ 4 ◯ 5 ◯ 6 ◯ 7 ◯ 8 ◯ 9 ◯ 10 (=absolutely necessary)

Stethoscope

◯ 1 (=no importance) ◯ 2 ◯ 3 ◯ 4 ◯ 5 ◯ 6 ◯ 7 ◯ 8 ◯ 9 ◯ 10 (=absolutely necessary)

Mucous membrane color and CRT

◯ 1 (=no importance) ◯ 2 ◯ 3 ◯ 4 ◯ 5 ◯ 6 ◯ 7 ◯ 8 ◯ 9 ◯ 10 (=absolutely necessary)

Section 9: Defibrillation

Do you have an electrical defibrillator available?

◯ Yes
◯ No

Which of the following techniques have you employed during CPR?

Check all that apply.

☐ External defibrillation
☐ Internal defibrillation
☐ Precordial thump
☐ None of the above

Section 10: CPR drugs

Which of the following drugs do you have readily available during CPR?

Check all that apply.

- ☐ Atropine
- ☐ Epinephrine (= adrenaline)
- ☐ Vasopressin
- ☐ Lidocaine
- ☐ Amiodarone
- ☐ Sodium bicarbonate
- ☐ Calcium gluconate
- ☐ Doxapram
- ☐ Mannitol
- ☐ Glucocorticoids
- ☐ Other

Please specify

____

Which of the following drugs have you used during CPR attempts in the last two (2) years?

Check all that apply.

- ☐ Atropine
- ☐ Epinephrine (=adrenaline)
- ☐ Vasopressin
- ☐ Lidocaine
- ☐ Amiodarone
- ☐ Sodium bicarbonate
- ☐ Calcium gluconate
- ☐ Doxapram
- ☐ Mannitol
- ☐ Glucocorticoids
- ☐ Other

Please specify

____

Do you routinely use intravascular volume expansion therapy (e.g., crystalloid or colloid bolus or boluses) as part of your CPR strategy?

- ◯ Yes
- ◯ No

Section 11: Route of drug administration

How frequently have you personally used the methods listed below for drug administration during CPR?

Intravenous drug administration

◯ 1 (=never) ◯ 2 ◯ 3 ◯ 4 ◯ 5 ◯ 6 ◯ 7 ◯ 8 ◯ 9 ◯ 10 (=always)

Intraosseous administration in a juvenile animal

◯ 1 (=never) ◯ 2 ◯ 3 ◯ 4 ◯ 5 ◯ 6 ◯ 7 ◯ 8 ◯ 9 ◯ 10 (=always)

Intraosseous administration in an adult animal

◯ 1 (=never) ◯ 2 ◯ 3 ◯ 4 ◯ 5 ◯ 6 ◯ 7 ◯ 8 ◯ 9 ◯ 10 (=always)

Direct (blind) intracardiac injection

◯ 1 (=never) ◯ 2 ◯ 3 ◯ 4 ◯ 5 ◯ 6 ◯ 7 ◯ 8 ◯ 9 ◯ 10 (=always)

Via endotracheal tube

◯ 1 (=never) ◯ 2 ◯ 3 ◯ 4 ◯ 5 ◯ 6 ◯ 7 ◯ 8 ◯ 9 ◯ 10 (=always)

Section 12: Your opinion on CPR and CPR guidelines

Conducting effective CPR is an essential skill in clinical small animal veterinary medicine.

○ Strongly disagree ○ Disagree ○ No opinion ○ Agree ○ Strongly agree

How do you judge your proficiency in performing CPR?

○ Poor ○ Fair ○ Good ○ Very good ○ Excellent

Have you heard of the Reassessment Campaign on Veterinary Resuscitation (RECOVER) CPR consensus guidelines published in 2012?

○ Yes
○ No

Have you consulted the RECOVER guidelines for direction and updates in performing CPR in your patients?

○ Yes
○ No

Does your workplace use any of the following RECOVER cognitive aid charts?

Check all that apply.

☐ CPR Algorithm
☐ CPR Emergency Drugs and Doses
☐ Post-Cardiac Arrest Algorithm
☐ None of the above
☐ I don't know

How useful do you rate each of the charts?

CPR Algorithm

○ 1 (=not useful) ○ 2 ○ 3 ○ 4 ○ 5 ○ 6 ○ 7 ○ 8 ○ 9 ○ 10 (=extremely useful)
○ Can't say, I do not know it

CPR Emergency Drugs and Doses

○ 1 (=not useful) ○ 2 ○ 3 ○ 4 ○ 5 ○ 6 ○ 7 ○ 8 ○ 9 ○ 10 (=extremely useful)
○ Can't say, I do not know it

Post-Cardiac Arrest Care Algorithm

○ 1 (=not useful) ○ 2 ○ 3 ○ 4 ○ 5 ○ 6 ○ 7 ○ 8 ○ 9 ○ 10 (=extremely useful)
○ Can't say, I do not know it

Please rate the following statements in relation to YOUR PERSONAL EXPERIENCE with the RECOVER guidelines.

"In my experience, the use of the RECOVER guidelines......

.....promoted CPR training."

○ Strongly disagree ○ Disagree ○ No opinion ○ Agree ○ Strongly agree

.....improved CPA recognition and initiation of effective CPR."

○ Strongly disagree ○ Disagree ○ No opinion ○ Agree ○ Strongly agree

.....improved quality of basic life support (e.g., chest compressions, ventilatory support)."

○ Strongly disagree ○ Disagree ○ No opinion ○ Agree ○ Strongly agree

.....improved quality of advanced life support (e.g., drug administration, defibrillation)."

◯ Strongly disagree ◯ Disagree ◯ No opinion ◯ Agree ◯ Strongly agree

....increased return of spontaneous circulation (ROSC) rates."

◯ Strongly disagree ◯ Disagree ◯ No opinion ◯ Agree ◯ Strongly agree

....increased survival to hospital discharge rates."

◯ Strongly disagree ◯ Disagree ◯ No opinion ◯ Agree ◯ Strongly agree
